# Testing the Associations Between Adult Playfulness and Sensation Seeking: A SEM Analysis of Librarians and Police Officers

**DOI:** 10.3389/fpsyg.2021.667165

**Published:** 2021-06-04

**Authors:** Kay Brauer, Tiziana Scherrer, René T. Proyer

**Affiliations:** Department of Psychology, Martin Luther University Halle-Wittenberg, Halle (Saale), Germany

**Keywords:** adult playfulness, librarians, OLIW, police, sensation seeking, measurement invariance

## Abstract

Playfulness is an understudied personality trait in adults. We examined the relationships between facets of adult playfulness and sensation seeking (SS) in distant vocational groups, namely, librarians (*N* = 339) and police officers (*N* = 399). First, manifest and latent group comparisons (measurement invariance [MI] analysis) showed that police officers were higher in SS than librarians, while we found no group differences for playfulness. Second, structural equation modeling (SEM) analyses showed that playfulness was widely positively related to SS, and findings were replicated across groups. However, the effects were of small to moderate size, and playfulness and SS shared between 4 and 22% variance. Our findings indicate that playfulness is not redundant with SS. Our study extends the understanding of adult playfulness by clarifying its overlap and distinctiveness from SS.

## Introduction

While playfulness is well-studied in children, there is increasing interest in the structure and consequences of playfulness in adults [see Bittermann et al. (2021)]^1^. Despite behavioral similarities such as risk-taking, the search for novel impressions, and stimulation (e.g., Zuckerman, 1994; Panksepp, 1998; Proyer, 2017; Proyer et al., 2019b), no study has yet examined its associations with sensation seeking (SS). We aimed to narrow this gap in the literature by testing the relationships between dimensions of playfulness and SS in subgroups of professions typically considered low (librarians) and high in SS (police officers).

### Adult Playfulness

A recent definition of playfulness in adults is: “[…] an individual differences variable that allows people to frame or reframe everyday situations in a way such that they experience them as entertaining, and/or intellectually stimulating, and/or personally interesting.” (Proyer, 2017, p. 114). This definition is accompanied by a multidimensional structural model of adult playfulness: the OLIW model, which consists of *Other-directed* (e.g., using playfulness in social situations to solve tension), *Lighthearted* (e.g., a spontaneous view of life without thinking much of consequences of the behavior; liking improvising over planning), *Intellectual* (e.g., enjoying play with new ideas and come up with new solutions for intellectual problems), and *Whimsical* playfulness (e.g., preferring odd or extraordinary things or people; Proyer, 2017). The OLIW facets show good observability in interpersonal perception studies testing the self-other agreement and consensus among observers (e.g., Proyer, 2017; Proyer and Brauer, 2018), and relate to numerous outcomes such as subjective well-being, mental and physical health (e.g., Proyer et al., 2018b; Farley et al., 2020), personality pathology (e.g., Proyer et al., 2020), and domains of romantic life [e.g., assortative mating, relationship satisfaction in actors and partners, or sexual role play; see e.g., Chick, 2001; Aune and Wong, 2002; Chick et al., 2012; Turley et al., 2017; Proyer et al., 2018a, 2019a; for an overview, see Brauer et al. (2021)]. The associations and overlap between playfulness and preferences in seeking stimulation by means of novel experiences have been discussed in the literature (e.g., Zuckerman, 1991, 1994; Panksepp, 1998; Zaleskiwicz, 2001), but its relationship with SS is not yet understood; our study aims to narrow this gap in the literature.

### Sensation Seeking

Zuckerman (1971, 1994) described SS as “a trait defined by the seeking of varied, novel, complex, and intense sensations and experiences, and the willingness to take physical, social, legal, and financial risks for the sake of such experience” (p. 27) and suggested a four-dimensional model comprising *Thrill and Adventure Seeking* (TAS; i.e., seeking for activities that include speed or danger), *Experience Seeking* (ES; i.e., seeking for mental experiences, travel, and non-conforming lifestyle), *Disinhibition* (i.e., seeking for social and sexual disinhibition, e.g., collective drinking, having many different sexual partners), and *Boredom Susceptibility* (BS; i.e., aversion against routine or repetition, expressed by restlessness when faced with monotony). The localization of SS in the Big Five showed robust positive relationships with Openness to Experience (e.g., Roberti, 2004). Further, extensive work on the physiological bases (e.g., hormones and neurotransmitters) and psychophysiological reactions (e.g., heart rate) has extended the understanding of SS by uncovering underlying physiological correlates (e.g., Zuckerman, 1979a, 1990, 1994; Zuckerman and Buchsbaum, 1980; Ballenger et al., 1983; Panksepp, 1998). SS relates to numerous behaviors and outcomes. For example, the literature provides robust evidence for the predictive value of SS for risk appraisals and perceptions (e.g., Zuckerman, 1991, 2007), behaviors such as substance use (e.g., Baker and Yardley, 2002; Martín-Fernández et al., 2019), hobbies (e.g., Furnham, 2004), vocational choices (e.g., Roth, 2003), gambling and sexual behaviors (e.g., Kalichman and Rompa, 1995; Reardon et al., 2019; Moynihan et al., 2021), and narrow domains such as political violence (Schumpe et al., 2020), to name but a few [for an in-depth overview, see Roberti (2004)].

### The Present Study

We aimed to study the differential associations between dimensions of playfulness and SS in adults. For the latter, we used two models, namely, Zuckerman's (1994) standard four-factor model, assessed with the *Sensation Seeking Scale*-V (SSS-V), and Arnett's (1994) two-factor model, assessed with the *Arnett Inventory of Sensation Seeking* (AISS). While Zuckerman's SSS-V assesses inclinations to novelty and *complexity* of stimulation, Arnett distinguishes between the need for *novelty* and *intensity* of stimulation. Ferrando and Chico (2001) have tested the overlap between both measures and summarize: “Most of the SSS items address behaviors related to activities of risk and focus on the complexity of the stimulatory situation, whereas the AISS items address the desire for seeking intensity and novelty in sensory experience.” (p. 1,122). However, their latent analyses of the instruments' structures and item characteristics have shown that both capture the higher-order SS trait, but that their dimensions are not redundant. Using both models allows us to examine the full range of SS in its relationship with playfulness.

Zuckerman (1979b, 1994) provides evidence for a positive relationship between playfulness and SS, for example, by using the adjective “playful” as an indicator of SS in early instruments to assess SS. Furthermore, initial findings on the overlap between inclinations to play(fulness) can be found in studies that examined the *Need for Play* (Murray, 1938), assessed with Jackson's (1974) *Personality Research Form* (PRF). When Zuckerman (1979a) examined the relationships between the PRF and his SSS, he found robust positive associations between all subscales and the Need for Play scale (*r*s ≤ 0.64), with Disinhibition showing the numerically highest correlation coefficients. Furthermore, Emmons et al. (1985) examined individual differences in leisure activities and found that the cluster representing “arousal behaviors” (e.g., “skiing” and “attending rock concerts”) positively correlated with the PRF's Need for Play scale (*r* = 0.40). While these findings provided early insights into the positive relationships between inclinations to play(fulness) and SS, it must be noted that Murray's definition of the Need for Play is (a) not meant as a definition of the personality trait and (b) somewhat limited in comparison with how playfulness is understood in current models. It only covers a hedonic orientation^2^, but does not, for example, encompass interpersonal or intellectual components (see e.g., Barnett, 2007; Proyer, 2017). Zuckerman (1994) argued that the correlations between SS and the Need for Play might be based on the shared element of seeking sensations of pleasure, but not necessarily seeking novel input and curiosity. The notion of “fun” or pleasure is common in many models of playfulness, but it has been acknowledged that playfulness also has other facets and, thus, it is worthwhile to examine other communalities between playfulness and SS. Nevertheless, these early studies provide support for the notion that a further examination of the association is of importance.

In our study, we expected positive associations between playfulness and SS. First, one might argue that engaging in the reframing process that characterizes playfulness (Proyer, 2017) is a means of decreasing monotony and boredom and creating novelty and sensation in one's environment. Further, laypeople perceive playfulness as a means to “experience variety, new things, and adventures,” “prevent routine,” “explore things,” and “spend time in a more interesting way” (Proyer, 2014). Barnett (2007) found that self- and other-rated playfulness relates robustly positive to being characterized as “adventurous” and “unpredictable.” Thus, the findings hint at positive relations between playfulness and SS, as those high in playfulness appear to be inclined to seek novelty and stimulation. This may be a by-product of broadening the understanding of what playfulness is in recent years (e.g., Barnett, 2007; Shen et al., 2014; Proyer, 2017).

Considering the OLIW facets, those high in Lighthearted playfulness prefer improvisation over planning with low considerations of consequences and Whimsical types of playfulness prefer unusual or odd things or people, and it could be argued that this reflects the desire for novelty and low monotony in those high in Lighthearted and/or Whimsical playfulness (Proyer, 2017), which could speak for a positive association with BS. Moreover, there is evidence that playfulness goes along with cognitive, social, and behavioral spontaneity, inclinations to impulsivity, and thinking styles that are denoted by novelty and low planning (e.g., Lieberman, 1977; Proyer, 2017; Proyer et al., 2019b). In line with such findings, the localization of the OLIW facets in a classification system of maladaptive personality traits (PID-5; Krueger et al., 2012) showed that Whimsical playfulness is related robustly to the Disinhibition domain, which is, among others, characterized by risk-taking (Proyer et al., 2020). Playfulness relates to exploration behaviors among humans and animals (e.g., Weisler and McCall, 1976; Lieberman, 1977; Panksepp, 1998; Shen, 2020), and Singer and Rummo (1973) found that children's playfulness is positively related to behaviors coded as novelty seeking/curiosity. The need for sensation can be satisfied by exploring new situations (especially as described in ES and TAS). Therefore, exploration could be a commonality of playfulness and SS.

Second, SS and playfulness might share a neurobiological basis. While Zuckerman [(1994); see also Panksepp (1998), Panksepp (2005), and Siviy (2016)] found that SS is linked to the activation in the dopaminergic system, there is good evidence for distinct activation patterns (i.e., different populations of dopaminergic neurons) in the same dopaminergic system in mammalians when they show playful behaviors [for a review, see Sivjy and Panksepp (2011)]. It seems feasible that similar mechanisms found in mammalian animals could exist in humans. Thus, the notion that playfulness and SS might share overlap finds support in neurobiological structures and mechanisms.

We used a comparative approach by testing the playfulness–SS associations in two distant vocational groups, namely, librarians, and police officers. Prior evidence suggests that SS relates to vocational choices [i.e., high SS goes along with choosing high-risk jobs; see Roberti (2004) and Zuckerman (2007)]. For example, Musolino and Hershenson (1977) found that sensation seekers chose a career in police as high-risk job that is also denoted by a variety in stimulation, situations, and low predictability, whereas librarians chose a vocation that is characterized by low risk, structured tasks, low variability across situations, and predictability. Using distant groups would allow us to examine whether (a) the structure of playfulness and SS is the same in both groups or whether differences exist in one or both constructs, and (b) associations between facets of playfulness and SS are comparable across groups.

Taken together, our aim was twofold. First, we compared the groups concerning the structure and expressions in playfulness and SS using MI analysis. In line with prior findings (Roberti, 2004), we expected higher expressions in SS in police officers than in librarians, whereas we tested the group differences in playfulness exploratorily. We argue that potential differences in the findings contribute to understanding the overlap between the variables. If playfulness and SS are redundant, the same degree of invariance should exist for playfulness and SS across groups. Second, we examined the relationships between playfulness and SS by using a global (Proyer, 2012b) and multifaceted approach (OLIW; Proyer, 2017) to playfulness and two conceptions of SS (Zuckerman, 1971; Arnett, 1994). Using two extreme groups and two approaches to the study variables allowed us to compare the associations across samples and examine the stability and generalizability of the findings.

## Methods

### Participants

#### Librarians

The sample comprised 339 German-speaking participants between 18 and 67 years of age (*M* = 41.5; *SD* = 13.4). The gender ratio in our sample (90.3% females) is in line with the typical gender distribution in this vocational group, where ~85% of librarians are female (cf. Arbeitsgruppe Berliner Bibliothekare, 1995). The educational status was high, as 56.7% of participants held a university degree, 25.7% had a high school diploma allowing them to attend university, 17.6% had a degree from secondary school, and 7.0% were in vocational training. On average, they had worked for 17.1 years (*SD* = 13.1; [0.0 and 46.0]) as librarians.

#### Police

This sample comprised 399 German police officers (46.0% female) between 19 and 60 years of age (*M* = 31.9; *SD* = 8.9). The educational status was slightly lower than in Sample 1, as 39.5% of the participants held a university degree, 41.8% had a high school diploma allowing them to attend university, and 18.7% had a degree from secondary school. On average, they had worked for 11.4 years (*SD* = 9.4; [0.5, 44]). The detailed job positions are displayed in the ESM A.

### Instruments

The *Short Measure of Adult Playfulness* (SMAP; Proyer, 2012b) assesses global playfulness in the sense of an easy onset and frequent display of playful behaviors with five items. Responses are given on a 7-point Likert-type scale (1 = “*does not apply at all*;” 7 = “*applies completely*;” sample item: “I am a playful person”). Proyer (2012b) reported high reliability (α ≥ 0.80; test–retest correlation of 0.74 for up to 16 weeks). There is robust evidence for the convergent, discriminant, factorial, and predictive validity.

The *OLIW-Playfulness Questionnaire* (OLIW; Proyer, 2017) assesses four facets of playfulness with seven items each on a 7-point Likert-type scale (1 = “*does not apply at all*;” 7 = “*applies completely*”). The facets are *Other-directed* (e.g., “I can use my playfulness to bring joy to other people or cheer them up”), *Lighthearted* (e.g., “Many people take their lives too seriously; when things don't work, you just have to improvise”), *Intellectual* (e.g., “If I want to develop a new idea further and think about is, I like to do this is a playful manner”), and *Whimsical* (e.g., “I have the reputation of being somewhat unusual or flamboyant”). Proyer (2017) reported internal consistencies between 0.66 (*Other-directed*) and 0.78 (*Whimsical*) and retest correlations (≥0.67, ≤ 3 months), which are in line with expectations for short scales covering the breadth of an underlying construct (Ziegler et al., 2014). There is robust evidence for the validity of the instrument [e.g., self-ratings converge well with peer ratings, aggregated diary data across 14 days, and inter-rater agreement, see also Proyer and Brauer (2018)].

The AISS [Arnett, 1994; German version by Roth and Herzberg (2004)] assesses SS concerning the dimensions *Intensity* and *Novelty* with 10 items each. Also, a total score can be computed. Sample items are “When I listen to music, I like it to be loud” (*Intensity*) and “I would like to travel to places that are strange and far away” (*Novelty*), and responses are given on a 4-point Likert-type scale (1 = “*does not apply at all*;” 4 = “*applies completely*”). Internal consistencies were reported as acceptable (α ≥ 0.64; Roth and Herzberg, 2004) for comparatively short scales that aim to assess the breadth of a construct (Ziegler et al., 2014). The AISS showed factorial, convergent, discriminant, and predictive validity.

*The* SSS-V (Zuckerman, 1994; German version: Beauducel et al., 1999) assesses four facets of SS with 10 items per scale: TAS, ES, *Disinhibition*, and BS. The SSS-V uses the forced-choice response format (e.g., A: “I like to spend time in familiar surroundings at home”; B: “I get very anxious when I have to stay home for certain periods of time”; BS). Internal consistencies are on the low end of expectations (e.g., ≥ 0.46; Beauducel et al., 1999; Ferrando and Chico, 2001) but retest correlations were high (0.41–0.75 for 6–8 months; Zuckerman, 1994). The instrument showed convergent, discriminant, and predictive validity.

### Procedure

The librarians were recruited via mailing lists from the German librarian portal and via social media and police officers via local police labor unions, the interior ministries of the German federal states, the German police academy, and social media. All participants gave informed consent and completed all instruments online (*SoSci Survey*; www.soscisurvey.de). Participants were not financially compensated but received feedback upon request and could enter a lottery for one of four online shopping vouchers (25€ each). Both samples completed the same questionnaire, which took 20 min for completion. Our study was conducted in accordance with the World Medical Association's Declaration of Helsinki. The participants provided their informed consent to participate in this study. This type of study is exempt from ethics approval in Germany, as conducting psychological studies collecting questionnaire data are guided by the ethical guidelines of the German Psychological Association (https://www.dgps.de/fileadmin/documents/ethikrl2004.pdf). We adhered to these guidelines^3^.

### Data Analysis

First, we compared the mean scores of the playfulness and SS questionnaires between the librarians and police officers with manifest and latent approaches. We computed standardized effect sizes (Hedges' *g*; ≥ 0.20/0.50/0.80, indicating small/medium/large effects) for comparing the manifest scale means across librarians and police, and compared their latent structure using MI analysis (Cheung and Rensvold, 2002). We tested MI in a series of multigroup confirmatory factor analyses (CFA) by consecutively constraining parameters across the groups: (1) *configural invariance* assumes that the number of latent factors is equal across groups; (2) *metric invariance* exists when the item-factor loadings are equal across groups; and (3) *scalar invariance* exists when latent means (intercepts) are equal across groups. The decision for rejecting an MI model is based on global model comparisons according to the comparative fit index (CFI). Cheung and Rensvold (2002) recommend interpreting changes of ΔCFI ≤ −0.01 as a significant deviation between models (i.e., accepting the model with fewer restrictions). For transparency, we report the standard fit indexes for each step and their differences (Δ) between each step, namely, root mean square error of approximation (RMSEA), including its 90% confidence interval (CI), Tucker–Lewis index (TLI), χ^2^ values, the Akaike information criterion, and the Bayesian information criterion (BIC).

Second, we tested the associations between playfulness and SS by computing bivariate correlations, controlled for age and gender. To account for potential influences of the instruments' reliabilities, we also computed the associations between the latent variables using structural equation modeling (SEM; maximum-likelihood estimation). We computed regression analyses to identify which SS scales predicted^4^ the SMAP and OLIW scales after controlling for age and gender (method = enter; Step 1 = age and gender; Step 2 = SS scales). Also, we computed the shared variance between the dimensions of playfulness and SS by computing linear regressions with each SS scale as outcome and the OLIW facets as predictors (Step 1 = age and gender; Step 2 = OLIW scales; method = enter). We interpreted the regression effect size *f* ^2^ (≥ 0.02/0.15/0.35, indicating small/medium/large effects).

We computed the MI and SEM analyses in *Mplus* 8.3 (Muthén and Muthén, 2017). All materials, data, and syntaxes are openly available in the Open Science Framework (osf.io/rswd2/).

Power analyses (G^*^Power; Faul et al., 2009) showed that our sample sizes allowed us to detect small effect sizes of ρ = 0.15 (librarians) and 0.14 (police officers), with a 5% type I error rate and 80% power. The sample sizes meet the recommendations for SEM and MI analyses (e.g., Cheung and Rensvold, 2002).

## Results

### Preliminary Analyses

Descriptive statistics are displayed in Table 1. The internal consistencies for the SMAP and OLIW scales were comparable to prior studies (e.g., Proyer, 2017; α ≥ 0.71; except for Intellectual playfulness: 0.62 and 0.57 in librarians and police officers). The internal consistencies of the AISS were lower in comparison with previous findings (Roth and Herzberg, 2004), whereas the SSS-V subscales met expectations from the literature (0.42 ≤ α ≤ .78). The means and standard deviations of all measures were in line with prior studies in German-speaking samples (e.g., Beauducel et al., 1999; Roth and Herzberg, 2004; Proyer, 2017). The skewness (≤ |0.45|) and kurtosis ( ≤ |1.04|) indicated no deviations from the normal distribution in all instruments.

**Table 1:** Descriptive statistics and internal consistencies for the playfulness and sensation seeking scales.

	**Librarians (*****N*** **=** **339)**		**Police officers (*****N*** **=** **399)**		**Hedges' *g***
	***M* (*SD*)**	**α**	***M* (*SD*)**	**α**	
SMAP	4.13 (1.40)	0.92	4.10 (1.26)	0.89	−0.02
**OLIW**					
Other-directed	4.66 (0.96)	0.74	4.98 (0.84)	0.71	0.36
Lighthearted	4.03 (0.97)	0.79	4.06 (0.94)	0.75	0.03
Intellectual	4.24 (0.79)	0.62	3.97 (0.71)	0.57	−0.36
Whimsical	4.06 (0.96)	0.77	3.92 (0.88)	0.74	−0.15
**AISS**					
Novelty	2.57 (0.42)	0.45	2.72 (0.43)	0.52	0.35
Intensity	2.06 (0.40)	0.47	2.69 (0.46)	0.60	1.45
Total	2.32 (0.34)	0.59	2.66 (0.37)	0.67	0.95
**SSS-V**					
TAS	1.40 (0.29)	0.78	1.65 (0.27)	0.78	0.90
ES	1.55 (0.18)	0.50	1.53 (0.17)	0.44	−0.12
DIS	1.26 (0.19)	0.53	1.41 (0.22)	0.58	0.73
BS	1.32 (0.19)	0.50	1.40 (0.18)	0.42	0.43
Total	1.38 (0.14)	0.78	1.50 (0.14)	0.75	0.86

*SMAP, Short Measure of Adult Playfulness; AISS, Arnett Inventory of Sensation Seeking; TAS, Thrill and Adventure Seeking; ES, Experience Seeking; DIS, Disinhibition; BS, Boredom Susceptibility; SSS-V, Sensation Seeking Scale*.

The correlations between playfulness and SS with age and gender are displayed in ESM B. In both samples, younger age is related to higher scores in the SMAP and Other-directed playfulness (*r*s ≤ 0.38), and Disinhibition, TAS, and Intensity in Librarians (*r*s ≤ 0.39) while showing negligible effects for police officers (*r*^2^ ≤ 2.3%). The examination of gender effects showed that men had higher scores in Other-directed Playfulness (*g*s ≥ |0.28|) in both samples. Men also had higher scores in Lighthearted playfulness and SMAP (*g*s ≥ |0.24|) in the police officers. For SS, men were higher than women in the police sample (|0.20| ≤ *g*s ≤ |0.68|), whereas men had higher scores for Intensity, Disinhibition, and BS in librarians (*g*s ≥ |0.37|).

The comparison of librarians and police officers showed two robust mean differences in adult playfulness (Table 1); namely, librarians were lower in Other-directed playfulness but higher in Intellectual playfulness (*g*s = |0.36|). As expected, police officers were substantially higher in almost all facets of SS (0.35 ≤ *g* ≤ 1.45; exception: ES).

### Measurement Invariance

#### Playfulness

Table 2 gives the fit and change indexes for the models for the SMAP, OLIW, and AISS^5^. For the SMAP, we accepted the scalar invariance model (ΔCFI ≥ −0.006), indicating the same dimensionality, loadings, and latent means across librarians and police officers. For the OLIW, the MI analysis provided evidence for rejecting scalar invariance in the OLIW scales (ΔCFI = −0.038) and accepting the metric invariance model (ΔCFI = −0.004); that is, the dimensionality and item-factor loadings were equal among librarians and police officers, whereas their latent means differed. The latter is in line with our analyses of group differences using the manifest scale scores (see Table 1).

**Table 2:** Measurement invariance analysis of the SMAP, OLIW facets, and AISS dimensions in 339 librarians and 399 police officers.

		**Fit indices**			**Model comparisons**		
		**Configural invariance (I)**	**Metric invariance (II)**	**Scalar invariance (III)**	**ΔI vs. II**	**ΔII vs. III**	**ΔI vs. III**
SMAP	CFI	0.991	0.986	0.980	−0.005	−0.006	−0.011
	RMSEA	0.084	0.086	0.092	0.002	0.006	0.008
	90%-CI	0.05, 0.114	0.062, 0.112	0.071, 0.114	-	-	-
	TLI	0.982	0.981	0.978	−0.001	−0.003	−0.004
	χ^2^	35.695	52.193	73.773	16.498	21.580	38.078
	df (χ^2^)	10	14	18	4	4	8
	AIC	10909.479	10917.977	10931.557	8.498	13.580	22.078
	BIC	11047.516	11037.050	10962.926	10.466	74.124	84.590
OLIW	CFI	0.816	0.812	0.774	−0.004	−0.038	−0.042
	RMSEA	0.060	0.060	0.064	0.000	0.004	0.004
	90%-CI	0.056, 0.064	0.056, 0.063	0.061, 0.068	-	-	-
	TLI	0.797	0.800	0.768	−0.003	−0.032	−0.029
	χ^2^	1598.716	1641.472	1851.814	42.756	210.342	253.098
	df (χ^2^)	688	712	736	24	24	48
	AIC	69234.105	69228.860	69391.202	5.245	162.342	157.097
	BIC	70062.326	69946.652	69998.565	155.674	51.913	63.761
AISS	CFI	0.676	0.623	0.530	−0.053	−0.093	−0.146
	RMSEA	0.060	0.063	0.068	0.003	0.005	0.008
	90%-CI	0.053, 0.065	0.058, 0.068	0.063, 0.074	-	-	-
	TLI	0.636	0.598	0.522	−0.038	−0.076	−0.114
	df (χ^2^)	784.168	875.063	1021.364	90.895	146.301	237.196
	df	338	356	374	18	18	36
	AIC	40419.212	40474.060	40584.408	54.848	110.348	165.196
	BIC	40980.893	40952.870	40980.348	28.023	27.478	0.545

*SMAP, Short Measure of Adult Playfulness; OLIW, OLIW Measurement of Adult Playfulness; AISS, Arnett Inventory of Sensation Seeking; CFI, comparative fit index; RMSEA, root mean square error of approximation; TLI, Tucker–Lewis Index; df, degrees of freedom; AIC, Akaike information criterion; BIC, Bayes information criterion*.

#### Sensation Seeking

The inspection of the model fit for the AISS suggested rejecting metric invariance and accepting configural invariance (all ΔCFI ≤ −0.05). Thus, the latent AISS loading and mean structure differed between police officers and librarians (i.e., items are differentially related to the underlying factors). Thus, in addition to the mean differences found on the manifest level (Table 1), item-factor loadings vary between librarians and police officers.

### Associations Between Adult Playfulness and SS

The correlations between playfulness and SS are displayed in Table 3 (librarians) and Table 4 (police officers). Overall, the associations were in the expected positive direction, of small-to-medium effect sizes, and did not indicate redundancy between playfulness and SS. The OLIW facets explained between 4 (Intensity; *f* ^2^ = 0.05) and 23% (ES; *f* ^2^ = 0.30) in variation of librarians' SS and between 6% (Disinhibition; *f* ^2^ = 0.07) and 19% (ES; *f* ^2^ = 0.24) in police officers' SS after controlling for age and gender (see Table 5 column ▵*R*^2^).

**Table 3:** Associations between playfulness and sensation-seeking scales in 339 librarians in correlation (*r*) and SEM analyses (β).

		**AISS**			**SSS-V**				
		**Novelty**	**Intensity**	**Total**	**TAS**	**ES**	**DIS**	**BS**	**Total**
SMAP	*r*	0.14^*^	0.05	0.12^*^	0.15^**^	0.16^**^	0.04	0.10	0.17^**^
	β	0.20^**^	0.10	0.18^**^	0.18^**^	0.27^***^	0.04	0.21^**^	-
Other-directed	*r*	0.14^**^	0.15^**^	0.18^**^	0.22^***^	0.18^**^	0.07	0.08	0.21^***^
	β	0.26^***^	0.24^**^	0.26^***^	0.29^***^	0.35^***^	0.16^*^	0.36^***^	-
Lighthearted	*r*	0.29^***^	0.19^***^	0.30^***^	0.29^***^	0.36^***^	0.30^***^	0.31^***^	0.45^***^
	β	0.33^***^	0.15^*^	0.25^***^	0.36^***^	0.65^***^	0.37^***^	0.58^***^	0.54^***^
Intellectual	*r*	0.30^***^	0.10	0.25^***^	0.23^***^	0.33^***^	0.10	0.15^**^	0.30^***^
	β	0.33^***^	0.18^*^	0.25^***^	0.29^***^	0.49^***^	0.18^**^	0.40^***^	-
Whimsical	*r*	0.30^***^	0.09	0.25^***^	0.20^***^	0.39^***^	0.22^***^	0.27^***^	0.38^***^
	β	0.33^***^	0.09	0.23^**^	0.21^***^	0.60^***^	0.30^***^	0.11	-

*SMAP, Short Measure of Adult Playfulness; TAS, Thrill and Adventure Seeking; ES, Experience Seeking; DIS, Disinhibition; BS, Boredom Susceptibility*.

* *p < 0.05*;

** *p < 0.01*;

*** *p < 0.001*.

*Two-tailed. - = Could not be estimated*.

**Table 4:** Associations between playfulness and sensation-seeking scales in 399 police officers in correlation (*r*) and SEM analyses (β).

		**AISS**			**SSS-V**				
		**Novelty**	**Intensity**	**Total**	**TAS**	**ES**	**DIS**	**BS**	**Total**
SMAP	*r*	0.17^**^	0.21^***^	0.23^***^	0.25^***^	0.13^**^	0.17^**^	0.09	0.26^***^
	β	0.20^**^	0.21^***^	0.24^***^	0.27^***^	0.25^***^	0.20^***^	-	-
Other-directed	*r*	0.17^**^	0.16^**^	0.20^***^	0.22^***^	0.16^**^	0.21^***^	0.04	0.26^***^
	β	0.27^***^	0.26^***^	0.32^***^	0.30^***^	0.33^***^	0.34^***^	-	-
Lighthearted	*r*	0.30^***^	0.26^***^	0.35^***^	0.29^***^	0.35^***^	0.17^**^	0.27^***^	0.41^***^
	β	0.39^***^	0.30^***^	0.41^***^	0.38^***^	0.60^***^	0.24^***^	0.49^***^	-
Intellectual	*r*	0.23^***^	0.15^**^	0.23^***^	0.21^***^	0.32^***^	0.18^***^	0.17^**^	0.33^***^
	β	0.25^***^	0.17^**^	0.25^***^	0.24^***^	0.56^***^	0.31^***^	0.43^***^	-
Whimsical	*r*	0.27^***^	0.15^**^	0.26^***^	0.13^*^	0.31^***^	0.17^**^	0.30^***^	0.33^***^
	β	0.33^***^	0.24^***^	0.33^***^	0.14^*^	0.54^***^	0.18^**^	-	-

*SMAP, Short Measure of Adult Playfulness; TAS, Thrill and Adventure Seeking; ES, Experience Seeking; DIS, Disinhibition; BS, Boredom Susceptibility.*

* *p < 0.05*;

** *p < 0.01*;

*** *p < 0.001*.

*Two-tailed. - = Could not be estimated*.

**Table 5:** Regression analyses predicting playfulness by sensation seeking facets of the SSS and AISS.

	**SMAP**		**Other-directed**		**Lighthearted**		**Intellectual**		**Whimsical**		***R*^**2**^ (Δ*R*^**2**^)**
	**β**	***p***	**β**	***p***	**β**	***p***	**β**	***p***	**β**	***p***	
**Librarians**											
Age	−0.40	<0.001	−0.19	0.001	0.05	0.575	0.07	0.205	−0.08	0.154	
Gender	−0.11	0.031	−0.14	0.011	0.03	0.381	−0.08	0.139	−0.08	0.142	
TAS	0.11	0.083	0.16	0.017	0.13	0.040	0.11	0.108	0.03	0.609	0.14 (0.11)
ES	0.11	0.074	0.14	0.029	0.21	<0.001	0.23	<0.001	0.27	<0.001	0.24 (0.23)
DIS	−0.05	0.382	−0.05	0.392	0.11	0.580	−0.06	0.305	0.05	0.370	0.19 (0.11)
BS	0.05	0.322	0.02	0.675	0.17	0.002	0.06	0.289	0.15	0.007	0.15 (0.13)
Novelty	0.03	0.611	−0.02	0.757	0.06	0.373	0.14	0.026	0.12	0.059	0.16 (0.15)
Intensity	−0.04	0.544	0.07	0.278	0.01	0.856	−0.04	0.574	−0.07	0.302	0.21 (0.04)
*R*^2^ (Δ*R*^2^)	0.20 (0.04)		0.13 (0.06)		0.22 (0.22)		0.16 (0.14)		0.21 (0.19)		
**Police**											
Age	−0.13	0.010	−0.19	<0.001	0.13	0.008	0.10	0.050	0.10	0.037	
Gender	−0.07	0.212	−0.11	0.027	−0.02	0.691	−0.02	0.637	0.01	0.861	
TAS	0.17	0.006	0.15	0.014	0.13	0.021	0.10	0.103	−0.04	0.534	0.17 (0.10)
ES	0.02	0.738	0.07	0.210	0.21	<0.001	0.24	<0.001	0.20	<0.001	0.20 (0.19)
DIS	0.11	0.039	0.17	0.001	0.02	0.765	0.09	0.089	0.03	0.510	0.09 (0.06)
BS	−0.01	0.883	−0.07	0.186	0.15	0.003	0.06	0.280	0.21	<0.001	0.14 (0.13)
Novelty	0.04	0.459	0.04	0.476	0.08	0.167	0.04	0.544	0.12	0.037	0.17 (0.12)
Intensity	0.09	0.159	0.04	0.558	0.09	0.130	0.00	0.972	0.02	0.680	0.20 (0.07)
*R*^2^ (Δ*R*^2^)	0.13 (0.08)		0.15 (0.08)		0.22 (0.19)		0.14 (0.13)		0.17 (0.16)		

*N = 339 (librarians) and 399 (police officers)*.

*TAS, Thrill and Adventure Seeking; ES, Experience Seeking; DIS, Disinhibition; BS, Boredom Susceptibility; R^2^, determination coefficient (in rows: shared variance between OLIW facets and SS facets, including age and gender; in columns: shared variance between SS facets and playfulness, including age and gender). ΔR^2^, shared variance after controlling for age and gender between OLIW facets and SS facets in rows, and shared variance after controlling for age and gender between SS facets and playfulness in columns*.

Overall, SEM and correlation analyses converged well (see Tables 3, 4). However, the latent scores of the SSS-V global factor could not be estimated in both samples, and the same was true for the BS factor in police officers.

#### Global Playfulness

In librarians, SMAP scores existed independently from AISS Intensity (β = 0.10, *p* = 0.175) and Disinhibition (β = 0.04, *p* = 0.550), but related to aspects of seeking novel input (TAS and ES) and BS (β ≥ 0.10, *p*s ≤ 0.006). In police officers, the SMAP was related to all SS scales (β ≥ 0.20; *p*s ≤ 0.001). The regression analysis identified TAS (β ≥ 0.10, *p* = 0.006) and Disinhibition (β = 0.17, *p* = 0.039) as statistically significant predictors in the police officers, whereas none of the SS scales significantly predicted SMAP scores in the librarians (β ≤ 0.11, *p*s ≥ 0.074)^6^. Overall, the SMAP shared only small amounts of overlap with SS, namely, 4% (librarians; *f* ^2^ = 0.05) and 8% (police officers; *f* ^2^ = 0.09) after controlling for age and gender.

#### Other-Directed Playfulness

We found positive associations with all SS scales in librarians (β ≥ 0.24; *p*s ≤ 0.003; exception: Disinhibition; β = 0.16 *p* = 0.220) and police officers (β ≥ 0.26; *p*s < 0.001). The regression analyses showed that TAS entered the regression model in both samples (β = 0.16 and 0.15 in librarians and police; *p*s ≤ 0.017). Additionally, ES showed a significant effect in librarians (β = 0.14, *p* = 0.029) and Disinhibition entered the model in police officers (β = 0.17, *p* = 0.001). Overall, Other-directed playfulness shared between 6 (librarians; *f* ^2^ = 0.07) and 8% (police officers; *f* ^2^ = 0.09) variance beyond age and gender.

#### Lighthearted Playfulness

We found robust associations with all SS dimensions in librarians and police officers (0.24 ≤ β ≤ 0.65; *p* < 0.001), except for Intensity in the librarians (β = 0.15; *p* = 0.041). In both samples, three SS facets predicted Lighthearted playfulness, namely, ES (β*s* = 0.21, *p*s < 0.001), BS (librarians: β = 0.17, *p* = 0.002; police: β = 0.15, *p* = 0.003), and TAS (β*s* = 0.13, *p*s ≤ 0.040). Overall, the SS scales explained 22 (librarians; *f* ^2^ = 0.28) and 19% (police officers; *f* ^2^ = 0.24) of the variance in Lighthearted playfulness beyond age and gender.

#### Intellectual Playfulness

We found robust positive relations (β ≥ 0.25; *p*s < 0.001) with all SS scales in librarians, except for numerically weaker associations with Intensity (β = 0.18; *p* = 0.016) and Disinhibition (β = 0.18; *p* = 0.003). Similarly, the relationships with Intensity were low in the police officers (β = 0.17; *p* = 0.008), whereas the remaining SS scores were positively related to Intellectual playfulness (β ≥ 0.24; *p* < 0.001). In the regression analyses, ES was the numerically strongest predictor in both samples (librarians: β = 0.23; police: β = 0.24, *p* < 0.002). In the librarians, Novelty also yielded a small effect (β = 0.14, *p* = 0.026). The SS scales explained 14 (*f* ^2^ = 0.17) and 13% (*f* ^2^ = 0.15) of the variance in librarians and police officers after controlling for age and gender.

#### Whimsical Playfulness

Whimsical playfulness demonstrated robust relationships with the SS scales (β ≥ 0.21; *p* ≤ 0.001) in the librarians, except for Intensity (β = 0.09; *p* = 0.242) and Disinhibition (β = 0.11; *p* = 0.260). For the police officers, the relation between Whimsical playfulness and TAS (β = 0.14; *p* = 0.015) as well as Disinhibition (β = 0.18; *p* = 0.002) was numerically lower than the remaining SS scales (β ≥ 0.24; *p* < 0.001). When regressing Whimsical playfulness to the SS scales, ES (librarians: β = 0.27; police: β = 0.20, *p*s < 0.001) and BS (librarians: β = 0.15; police: β = 0.21, *p*s < 0.007) were numerically strong predictors in both samples. Further, Novelty yielded a small effect (β = 0.12) that reached statistical significance in police officers (*p* = 0.037), but not in librarians (*p* = 0.059). Controlling for age and gender, the SS scales explained 19 (*f* ^2^ = 0.24) and 16% (*f* ^2^ = 0.19) of the variance in Whimsical playfulness scores in librarians and police officers, respectively.

## Discussion

Our study provides an initial insight on how individual differences in playfulness are related to SS, using global and multidimensional approaches and testing vocational groups differing in their risk proneness (librarians and police officers; e.g., Roberti, 2004). MI analyses indicated that robust group differences concerning loadings and means exist for the AISS (configural invariance) and manifest mean difference analyses demonstrated that police officers were higher in SS (small-to-large effect sizes), with ES being the exception. This is in line with prior findings showing that sensation seekers chose riskier jobs [see Roberti (2004) and Zuckerman (2007)]. On the contrary, playfulness measures demonstrated metric (OLIW; i.e., invariant loadings) *and* scalar invariance (SMAP; i.e., invariant latent means), thus showing a distinct pattern from the SS dimensions. The inspection of mean differences showed negligible effects except for Other-directed (higher in police officers) and Intellectual playfulness (higher in librarians). One might argue that Other-directed playfulness is important for policework (e.g., solving interpersonal conflict, but also for managing relationships among colleagues and coping with stressors at the workplace); Intellectual playfulness could stimulate thinking styles (Proyer et al., 2019b) that allow librarians to approach their work while avoiding routine and monotony. The latter is a perceived function of playfulness among lay people (Proyer, 2014). However, when comparing the expressions with other samples (e.g., Brauer and Proyer, 2017; Proyer, 2017), each group was in a comparable range without showing particularly elevated expressions. Hence, while SS showed the expected differences across librarians and police officers, playfulness was equally expressed across groups. We argue that the differential patterns of group differences in manifest indicators and the latent structures of the playfulness and SS measures across the groups of librarians and police officers provide evidence that playfulness and SS are not redundant.

MI could not be estimated for the SSS-V. Beauducel et al. (1999) reported that the factor structure of the German-language SSS-V did not converge with the original's (e.g., dimensionality and cross-loadings), and it is unclear whether this might compromise MI analysis. Replication in non-German-speaking samples is desirable to clarify this issue.

We analyzed the associations between playfulness and SS using manifest and latent scores. Overall, the findings of both approaches converged well and showed the expected positive associations with small-to-medium effect sizes. The playfulness–SS associations were widely identical across librarians and police officers, with few exceptions (e.g., Intensity and Disinhibition showing negligible correlations in librarians). Thus, although there are differences in the levels of SS among librarians and police officers, the associations with playfulness replicated well across samples and demonstrated stability. Further, regression analyses demonstrated small-to-medium effect sizes, indicating a moderate overlap in variance between playfulness and SS, and, thus, no redundancy between playfulness and SS. Overall, our findings showed markedly smaller effects sizes for the association between playfulness and SS than prior research (Jackson, 1974; Emmons et al., 1985; Zuckerman, 1994). This can probably be attributed to a broadening of the understanding of what facets of playfulness could be (e.g., also covering intellectual and interpersonal facets).

Using two conceptualizations of SS (Arnett, 1994; Zuckerman, 1994) showed that playfulness overlaps with the desire to explore new situations and environments, as Zuckerman's (1994) dimension of ES, and partly TAS, were good predictors of playfulness. Similarly, we found numerically higher associations between playfulness and the AISS Novelty facet, whereas the need for *intense* stimulation was widely unrelated. In Zuckerman's model, intensity is best described by Disinhibition, which also showed comparatively low associations and was not predictive of playfulness in regression analyses (exception: small effect in police officers' global and Other-directed playfulness). First, this is in stark contrast to Zuckerman (1994), who found strong correlations between the hedonic-oriented Need for Play scale (Jackson, 1974) and the SSS facet Disinhibition. However, this is in line with prior findings about the independence (Other-directed and Intellectual playfulness) or negligible overlap with the Disinhibition domain of Krueger et al.'s (2012) personality inventory of maladaptive personality traits (Proyer et al., 2020). Second, this pattern fits well into laypeople's notions of playfulness, i.e., providing a means to explore new situations and preventing routine (e.g., Barnett, 2007; Proyer, 2014), and lends evidence to the notion of playfulness and SS sharing inclinations to exploration. Playfulness related to seeking stimulation by novel experiences might contribute to understanding prior findings, for example, why those high in playfulness enjoy cognitive stimulation (e.g., preferring abstract over simple art) and engage in more intense forms of sexual role play such as bondage, discipline, dominance and submission, and sadomasochism (e.g., Proyer, 2012a; Turley et al., 2017; Brauer et al., 2021). Moreover, BS was a statistically significant predictor of Lighthearted and Whimsical types of playfulness, supporting the notion that those types of playfulness are characterized by both preferring stimulation but also dismissing monotony and repetition [see Proyer (2017)]. Future studies might examine whether those types of playfulness are prone to behaviors that induce stimulation but might be harmful (e.g., substance abuse). While Zuckerman argued that the overlap between inclinations to play(fulness) and SS is based on communalities in seeking pleasure, our findings rather indicate that playfulness, as understood as an individual differences variable that also accounts for engaging in intellectual and interpersonal play (Proyer, 2017), shares a common ground with SS in seeking new experiences, novel stimuli, and curiosity.

The comparison of global and facet-wise approaches to playfulness has again shown the merit of differentiating among the OLIW facets. While the SMAP was comparatively weakly associated with SS, the OLIW facets allowed the localization of SS dimensions into the four-facet structure of playfulness (Proyer, 2017) and revealed differential associations with SS. In terms of effect sizes, it is notable that Other-directed playfulness shared only small amounts of variance with SS. One could argue that the interpersonal nature of this facet is rather unrelated to experiencing broad inclinations to seeking novel stimuli and experiences.

Overall, our data show that SS and playfulness are neither identical nor redundant, but that playfulness is related to exploration in adults and children alike (Singer and Rummo, 1973; Lieberman, 1977). Our findings might be seen as initial support for the notion that playfulness and SS might share a sense of exploration, but are not the same neurobiological systems (e.g., Panksepp, 1998, 2005; Sivjy and Panksepp, 2011; Siviy, 2016). While our self-report data support this notion, it would be desirable to further examine the distinctiveness of playfulness and SS on the neurophysiological level. Future research should extend the findings by assessing preferences for leisure and work activities to examine the differential and shared associations with playfulness and SS to examine their distinctive predictiveness. Also, more narrow facets of SS, for example, *sexual* SS (Kalichman and Rompa, 1995), might allow for further conclusions on the novelty seeking of playful adults in the interpersonal domain, particularly since recent studies have provided evidence that playfulness contributes to sexual satisfaction in actors and partners (Proyer et al., 2019a), sexual identity, and role play (Turley et al., 2017; Paasonen, 2018), and is also related to the number of short-term and long-term partnerships [de Moraes et al., 2021; see also Brauer et al. (2021)]. Further, partialing out shared variance with broad personality traits (e.g., Openness to Experience) might clarify the unique playfulness–SS associations. Moreover, it would be interesting to study atypical groups. For example, testing whether librarians high in SS can capitalize from their playfulness to reach high work satisfaction and productivity, or whether police officers can use their playfulness to better cope with routine tasks. This knowledge might open the door for workplace interventions that could help individuals who are faced with a work environment or tasks that do not fit their personality (interests, e.g., sensation seekers who frequently have to deal with paperwork). Recently, training to increase playfulness on the trait level and that to change work designs toward more playfulness have been positively evaluated (Proyer et al., 2021; Scharp et al., 2021). Future research might use such approaches to examine the relationships with SS (e.g., do changes in playfulness go along with changes in SS?), but also to increase the personality-job fit and thereby work satisfaction in applied contexts. As in our example of a sensation seeker who has to frequently deal with paperwork or other monotonous tasks, redesigning these in a playful way might make the tasks more interesting and appealing. Finally, we encourage future research to examine the unique predictive values of playfulness and SS for outcomes such as leisure activities, occupational choices, and potentially harming behaviors such as substance use.

### Limitations

Our sample of librarians comprised mainly women. While the gender ratio in our sample represents the distribution of this vocational group well (Arbeitsgruppe Berliner Bibliothekare, 1995), replication in more heterogeneous samples with regard to gender is important to generalize our findings. Further, we collected only self-reports. We expect that the inclusion of peer ratings or diary data would contribute to reducing the shared method variance (Campbell and Fiske, 1959) and provide incremental insight into the tested associations [see Proyer (2017) and Proyer et al. (2020)]. As prior studies indicated that playfulness and SS can be accurately perceived by others (e.g., peers), we expect incremental information by using other ratings (Angleitner et al., 2004; Proyer and Brauer, 2018; Proyer et al., 2020). Therefore, a replication of the MI analyses on the basis of *peer* ratings is desirable to cross-validate our findings across methods. Finally, the generalizability of our findings is limited as we only tested German-speaking participants; replication in other cultures is desirable to examine potential cultural differences in the expressions and associations of playfulness and SS (e.g., Zuckerman et al., 1978; Barnett, 2017; Pang and Proyer, 2018). Also, our samples were comparatively homogeneous regarding sociodemographic variables (e.g., high educational status). Thus, replication in more heterogeneous samples with regard to sociodemographic variables is desirable in future research. However, our findings contribute to the knowledge concerning the overlap and distinctiveness between adult playfulness and SS and are a fruitful starting point for further research.

## Data Availability Statement

The datasets and syntaxes presented in this study can be found openly available in the Open Science Framework under https://osf.io/rswd2/.

## Ethics Statement

Ethical review and approval was not required for the study on human participants in accordance with the local legislation and institutional requirements. The patients/participants provided their written informed consent to participate in this study.

## Author Contributions

KB, TS, and RP involved in conceptualization, writing of the original draft, and reviewed and edited the written manuscript. RP involved in data curation. KB and TS involved in formal analysis. KB involved in methodology. All authors contributed to the article and approved the submitted version.

## Conflict of Interest

The authors declare that the research was conducted in the absence of any commercial or financial relationships that could be construed as a potential conflict of interest.

## Footnotes

^1^Bittermann et al. found in their analysis of Twitter microblogs (“tweets”) that playfulness emerged as a “hot topic” among scholars in psychology.

^2^Murray (1938) defines the Need for Play as “Play (Playful attitude). To relax, amuse oneself, seek diversion and entertainment. To ‘have Fun,' to play games. To laugh, joke and be merry. To avoid serious tension.” (p. 83).

^3^Guidelines include informing participants about the purpose and duration of the study, providing contact information to the principal investigator, providing information that participation can quit at all times, informing about potential consequences if to be reasonably expected, informing about the contribution of the knowledge to the field, ensuring anonymity and confidentiality, and informing about monetary or otherwise compensation for participation.

^4^Note that we use the expressions “predict” and “predictor” in its technical sense to describe independent variables and their associations with playfulness. The usage of these words does not imply assumptions of causality.

^5^MI could not be estimated for the SSS-V. Independently of the estimation algorithm, the model did not converge.

^6^Following a reviewer's suggestion, we have computed semi-partial correlations between the OLIW facets and the SS scales. These converge strongly with the reported beta coefficients (mean difference = 0.006) and are displayed in the OSF.
