# Supplementary material for: Testing the Associations Between Adult Playfulness and Sensation Seeking: A SEM Analysis of Librarians and Police Officers
Source: Front Psychol. 2021 Jun 4;12:667165. doi: 10.3389/fpsyg.2021.667165 (PMC8211874; doi:10.3389/fpsyg.2021.667165)
Supplement: Supplementary file 1 [file Data_Sheet_1.docx]

**Electronic Supplementary Material A**

*Relative Frequencies of Positions in the Sample of Police Officers*

| Post | Relative Frequency |
| --- | --- |
| Police Academy | 8.8% |
| Police Constable | 11.5% |
| Senior Police Constable | 15.0% |
| Police Sergeant | 10.0% |
| Junior Inspector | 31.1% |
| Inspector | 15.5% |
| Chief Inspector | 7.6% |
| Chief Superintendent | 0.3% |
| Senior Chief Superintendent | 0.3% |

**Electronic Supplementary Material B**

*Correlations Between Playfulness and Sensation Seeking Instruments With Age and Gender*

|  | Librarians | | | Police | | |
| --- | --- | --- | --- | --- | --- | --- |
|  | *r*_Age_ | *r*_Gender_ | *g* | *r*_Age_ | *r*_Gender_ | *g* |
| SMAP | -.38*** | -.06 | -0.20 | -.16** | -.12* | -0.24 |
|  |  |  |  |  |  |  |
| Other-directed | -.21*** | -.12* | -0.41 | -.20*** | -.14** | -0.28 |
| Lighthearted | -.01 | -.03 | -0.10 | .12* | -.12* | -0.25 |
| Intellectual | .08 | -.10 | -0.33 | .11* | -.08 | -0.17 |
| Whimsical | -.07 | -.11* | -0.04 | -.11* | -.06 | -0.13 |
|  |  |  |  |  |  |  |
| Novelty | -.10 | .00 | -0.01 | .03 | -.22*** | -0.45 |
| Intensity | -.39*** | -.11* | -0.37 | -.14** | -.31*** | -0.65 |
| AISS (Total) | -.29*** | -.07 | -0.23 | -.07 | -.32*** | -0.68 |
|  |  |  |  |  |  |  |
| TAS | -.18** | .06 | 0.20 | -.15** | -.18*** | -0.36 |
| ES | -.01 | -.09 | -0.31 | -.12* | -.01 | -0.02 |
| DIS | -.20*** | -.17** | -0.59 | -.09 | -.11* | -0.22 |
| BS | .02 | -.12* | -0.37 | -.07 | -.10* | -0.20 |
| SSS-V (Total) | -.15** | -.10 | -0.32 | -.09 | -.16** | -0.34 |

*Note.* Gender is coded 1= male, 2 = female. SMAP = Short Measure of Adult Playfulness. AISS = Arnett Inventory of Sensation Seeking. TAS = Thrill and Adventure Seeking. ES = Experience Seeking. DIS = Disinhibition. BS = Boredom Susceptibility. SSS-V = Sensation Seeking Scale. *g* = Hedges’ effect size for group comparison between gender. **p* < .05. ***p* < .01 ****p*< .001. Two-tailed.
